# Simple Gingivitis: Its Etiology and Treatment

**Published:** 1901-12

**Authors:** George T. Carpenter

**Affiliations:** Chicago, Ill.


### SIMPLE GINGIVITIS: ITS ETIOLOGY AND TREATMENT.[1]

BY GEORGE T. CARPENTER, M.D., CHICAGO, ILL.

DISEASE of the human gums in these days of advanced civilization is very common and almost universal. In fact, it is a rare thing to find gums that are in all their margins perfectly free from irritation, inflammation, hypertrophy, atrophy, or absorption, and many will show evidence of gingival ulceration. At three previous meetings of this section I have presented papers closely con-

[1] Abstract of a paper read before the American Medical Association, Section on Stomatology, St. Paul, Minn., June 4, 5, 6, and 7, 1901.

nected with the present subject. The conditions then studied were the result of advanced inflammatory or suppurative processes; but by the term "Simple Gingivitis" I include only that condition of the gum margin about the necks of the teeth known as gingiva, that shows the slightest departure from health but is fully established and persistent in its nature. It is the purpose of this paper to take gingival irritation in this simplest form and point out its etiology and treatment, and in this way prevent the subsequent and more serious diseases, which by their certain progress result in pain, discomfort, loss of tissue, and eventually the loss of the teeth themselves. Gingival irritation is liable to present itself at any point where there is a gingival margin. We may find the gingiva of one tooth inflamed and the rest of the gums in a healthy condition. We may find the anterior teeth in a clean condition but affected by gingivitis, while the gums of the posterior teeth, which receive less care, may be in a good healthy condition. We may find gingivitis in some or all of the gums in well-kept mouths, and we may find exactly the same condition of gums in mouths that do not receive the slightest care or attention. We will find this form of inflammation in the mouths of the young, those of middle life, and of old age. We will also find it in the anæmic and emaciated, also in the well-nourished and rugged. This condition, which is found in the human mouth, is rarely found in the mouths of lower animals in their natural state, and from some experiments on the gingiva of rabbits I find that it is almost impossible to establish a gingival irritation without using some powerful infectious or poisonous substance. We also find that in the human mouth where bands, clamps, wedges, or ligatures have caused some irritation, and even laceration, that a gingivitis does not, as a rule, result, so that the etiology of this apparently slight trouble is varied and very obscure. The etiology, or local causes, irregularities, and malocclusion are factors; also imperfect, or loss of, contour, improper use of the teeth, or, more correctly, insufficient use of the teeth, causing lack of tone to the gum; inorganic substances used as dentifrice, causing unnatural or insoluble deposits under gum margins, which is most common in the lower jaw; the injudicious use of toothpicks, floss, and rubber bands, keeping constant irritation at given points.

*Constitutional Causes.*—A defect in nutrition or cachectic condition may cause an isolated gingivitis at a given point, just as the same condition may produce an aphtha ulcer at any point on the mucous membrane of the mouth. Improper nourishment, which is the result of too much preparation of food for the teeth and stomach. Plain, coarse, wholesome food that will give the teeth something to do will act as a proper stimulus to the gums and will be kindly received by the stomach. The colored people at the close of the Civil War had mouths with healthy gums and teeth free from caries, but higher civilization, mingled with hotel and restaurant cooking, has so changed conditions that their mouths now are filled with both caries and disease. Careful examination of the mouths of many of the peasants emigrating from Europe have led me to believe that the care of the mouth should be principally through the stomach and a thorough use of the teeth by mastication of food.

*Treatment.*—Correct all malocclusions and restore perfect contour to crowns. Remove all local irritants and deposits and make a light application of tincture of iodine to affected parts, and give instruction to prevent irritation and abuse of gums; change diet from mashed vegetables of all descriptions, or soaked or cooked in milk, for dry or Swedish toast, whole wheat, or grape-nuts to grind thoroughly fine with the teeth, incorporate with the saliva, and swallow. This treatment should be followed, especially with children suffering from gingivitis.

I am indebted to Dr. M. H. Fletcher, of Cincinnati, for the suggestion of cornmeal as a dentifrice. I think, as a partial substitute for thorough mastication, that a good cereal dentifrice is very beneficial, using corn, oats, cream of wheat, or any coarse and grainy substance, and brush the teeth and gums vigorously after each meal and before retiring, giving a half rotation to the brush. The mouth should be thoroughly rinsed after each brushing, as there is some difficulty in removing the cereal. I do not approve of the use of a very stiff brush without a dentifrice, and I consider the place for all inorganic dentifrices is on the outside of the mouth. They may serve a good purpose for cleansing metals and marble, but should not be used on organized tissues.

